# On the Use of a Feedback Tracking Architecture for Satellite Navigation Spoofing Detection

**DOI:** 10.3390/s16122051

**Published:** 2016-12-02

**Authors:** Esteban Garbin Manfredini, Fabio Dovis

**Affiliations:** Department of Electronics and Telecommunications, Politecnico di Torino, Torino 10129, Italy; fabio.dovis@polito.it

**Keywords:** signal processing, GNSS receiver, spoofing detection, classification algorithm

## Abstract

In this paper, the Extended Coupled Amplitude Delay Lock Loop (ECADLL) architecture, previously introduced as a solution able to deal with a multipath environment, is revisited and improved to tailor it to spoofing detection purposes. Exploiting a properly-defined decision algorithm, the architecture is able to effectively detect a spoofer attack, as well as distinguish it from other kinds of interference events. The new algorithm is used to classify them according to their characteristics. We also introduce the use of a ratio metric detector in order to reduce the detection latency and the computational load of the architecture.

## 1. Introduction

The increasing concern for security in all electronic and telecommunication systems has affected many different sectors of today’s society, one of them being the Global Navigation Satellite Systems (GNSS). Modern society strongly relies on GNSS, for a constantly increasing number of applications and services. However, the issues related to the security of such systems is sometimes underestimated. This is the case of some services relying on GNSS civil signals. In fact, the menace of intentional radio-frequency interference, such as jamming or spoofing attacks, is gaining momentum, and discussions are being held all over the world trying to find ways to protect GNSS civil users from these attacks.

Nowadays, the effects of these intentional interferences, which are able to compromise the correct working of the GNSS receivers, are well known [1,2,3,4,5], and the need for improving the security of the receiver has been demonstrated [6,7], especially in the case of applications whose malfunctioning would put people’s safety at risk. Not only navigation and transportation companies often rely on the position obtained from the GNSS signals, but also power grids, telecommunications towers, banking procedures, etc., use GNSS to synchronize many different processes [3].

There are different types of intentional interference [5,8] that affect the correct functioning of GNSS receivers [3,5,9,10]. The most subtle and dangerous type of intentional interference is the spoofing attack, which consists of the transmission of GNSS-like signals, aiming at taking control of the receiver. If the spoofer takes control of the receiver, it is able to freely change its position and timing solutions without the receiver noticing any unexpected effect [3,9]. Spoofing attacks have been demonstrated on several occasions [6,11], and this has raised an alarm throughout the GNSS community. Different kinds of implementations of the spoofing attacks will be presented in Section 2.

In order to design a countermeasure to spoofing attacks, this paper revisits and improves a tracking technique known as Extended Coupled Amplitude Delay Lock Loop (ECADLL), originally presented in [12,13]. The goal of the work is to tailor the architecture to spoofing detection, making it also able to mitigate the effects created by spoofing attacks. Moreover, an effective algorithm for spoofer detection with the capability to distinguish between multipath and a spoofing attack is defined. The ECADLL was tested against realistic spoofing scenarios, and the performance was compared against another recently proposed anti-spoofing technique, known as the Signal Quality Monitoring Technique (SQMT) [14]. With the introduction of a ratio metric detection algorithm in the monitoring block, the detection latency was reduced, and the computational load of the algorithm was decreased.

The ECADLL consists of a feedback tracking architecture that was proposed as a multipath mitigation technique [12,13] able to remove multipath signals from the tracking channels. Given the similar effects that spoofing signals and multipath signals have when observed in the correlation domain, the ECADLL was later proposed as a spoofing detection technique [15,16]. However, a proper detection algorithm was never been detailed and tested in realistic scenarios. In the present study, we address these two unresolved aspects.

The paper is organized as follows: first, in Section 2, a review of the types of spoofing attacks and a recall of the most common anti-spoofing techniques are presented. In Section 3, the theoretical description of the working procedure of the ECADLL is revisited. Section 4 defines the spoofing detection algorithm, and an analytic explanation on how to better exploit the information provided by the ECADLL is provided. In Section 5, the experimental setup and the different scenarios used to obtain the results are described. In Section 6, results using the ECADLL are shown, demonstrating the spoofing detection capabilities of the algorithm and comparing them to another signal processing anti-spoofing technique. Finally, in Section 7, the conclusions of the work are drawn.

## 2. Types of Spoofing Attacks and Countermeasures

In recent research, the different types of spoofing attacks have been classified based on how the attack is constructed and on the difficulty of detecting it by a receiver point of view [9,17].

A simplistic spoofing attack is based on the use of a signal simulator to build the fake signal and re-transmit it in order to fool the receiver. This type of attack is very simple to realize, but it is also expensive and easily detectable by means of trivial techniques, given that a large signal power is needed and that the fake signal is not synchronized with the constellation.

The second type is known as intermediate attacks or receiver-based spoofing attacks. This type of spoofer has a built-in receiver that collects and tracks the satellite signal parameters, in order to generate a new signal that is consistent with the current constellation [11] and transmits it to the target receiver.

The third type of attack is called the sophisticated receiver-based spoofer. It aims to overcome one weakness of the intermediate attack, which is that it only broadcast from a single antenna and direction, thus the sophisticated version uses several different antennas to broadcast each satellite signal in order to be undetectable through anti-spoofing techniques that rely on angle of arrival discrimination. However, these attacks have a much higher complexity level than the previous two types, making them very difficult to realize.

From the three types of spoofing attacks, the intermediate spoofer is the most feasible and probable to cause damage, so is the one we will focus on throughout this article.

Since the first major publication reporting the feasibility of building a spoofer with a software-defined receiver and low cost components [18], the GNSS community has been continuously developing different anti-spoofing techniques, aimed at defending against the malicious signals at different stages of the receiver. These anti-spoofing techniques have very different approaches, and they can be classified into three main groups [3,9]:

(a) Cryptographic and authentication techniques: These methods propose to authenticate the incoming signal, using signals from different receivers or frequencies (L1/L2). Alternatively, they exploit the transmission of the P(Y)secured channel in order to identify if the received signal is authentic or not [19,20,21].
(b) Antenna-aided techniques: These techniques are based on the wide spatial correlation that the satellite signal has with respect to the spoofer signal during an intermediate attack. These techniques are vastly different, ranging from angle of arrival detection [22,23], to the use of synthetic arrays [24] and beam forming techniques that are able to mitigate the attacks by pointing null lobes at the spoofing signal [25,26].
(c) Receiver-level signal processing techniques: These methods are based on signal processing techniques, which focus on aspects that differ between the spoofing signal and the satellite one. Many different techniques have been proposed in this category, such as: techniques based on signal power monitoring, either monitoring the carrier to noise ratio ($C/N_{0}$) [27], absolute in-channel power measurements [28] or relative power variations [29]. Other signal processing techniques are based on the distribution analysis of the correlator output [30]. There exist also signal quality monitoring techniques, which aim at detecting distortions in the correlation function via the use of ratio metric tests [31,32,33,34,35]. Other techniques are based on the time of arrival and other consistency checks. Techniques based on goodness-of-fit tests have been proposed [36] along with techniques based on Receiver Autonomous Integrity Monitoring (RAIM) discrimination algorithms [29,37]. Finally, techniques based on the multiple Delay Lock Loop (DLL) used for spoofing detection and mitigation [12,13,15,16] have been proposed.

This article focuses on ECADLL, which is a receiver-level signal processing anti-spoofing technique based on a multiple-DLL architecture.

## 3. The ECADLL

The working principle of the ECADLL is based on distinguishing and tracking separately the satellite signal and the impairment signal, i.e., spoofer or multipath. It uses an architecture made of several DLLs and a feedback loop to remove the extra components from the incoming signal and track each signal individually. The incoming satellite signal at the baseband level for a single satellite, denoted as s(t) and under impairment presence denoted as m(t), can be written as:

(1) $$s\left(t\right)=a_{0}D\left(t-\theta_{0}\right)c_{f}\left(t-\tau_{o}\right)e^{j(2\pi \left(f_{IF}+f_{D}\right)t+\theta_{0})}+m\left(t\right)$$

where,

(2) $$m\left(t\right)=D\left(t-\theta_{0}-\theta_{n}\right)\sum_{n=1}^{M}a_{n}c_{f}\left(t-\tau_{0}-\tau_{n}\right)e^{j(2\pi \left(f_{IF}+f_{D}\right)t+\theta_{0}+\theta_{n})}+n_{f}\left(t\right)$$

and:

- $c_{f}\left(t\right)$ is the spreading code of the signal
- $\tau_{0},a_{0}$ and $\theta_{0}$ are the satellite signal code delay, amplitude and carrier phase, respectively
- $\tau_{n},a_{n}$ and $\theta_{n}$ denote the code delay, amplitude and carrier phase of the n-th impairment ray with respect to the LOS
- $f_{IF}$ is the intermediate frequency of the front-end
- $f_{D}$ is the Doppler shift of the signal carrier
- D(t) is the navigation data information
- $n_{f}\left(t\right)$ is the Gaussian noise

In (1) and (2), we observe the overall signal that is used by the receiver after the intermediate frequency down-conversion and the radio-frequency front-end filtering. The term (1) shows the signal coming from the satellite, while (2) contains all possible impairment rays along with a model of the noise of the signal.

Throughout this paper and in general for spoofing detection, M=1 will be assumed, due to the fact that only one powerful ray is assumed to be present during a spoofing attack. Given that the spoofing signal has the same structure as the satellite signal, the ECADLL uses this intrinsic similarity to track each ray individually, using a special tracking structure referred to as the unit. Each unit consists of a unique DLL, plus a couple of Amplitude Lock Loops (ALL), detailed in [12], that aim at estimating the delay $\tau_{n}$, the amplitude $a_{n}$ and the phase $\theta_{n}$ of the different rays.

The input signal of the i-th unit will be:

(3) $$s_{i}\left(t\right)=s\left(t\right)-\sum_{n\neq i}^{N}{\hat{a}}_{n}\cdot c\left(t-{\hat{\tau }}_{n}\right)e^{j({\hat{\theta }}_{n})}$$

where ${\hat{\tau }}_{n},{\hat{a}}_{n}$ and ${\hat{\theta }}_{n}$ are the estimated values obtained from the tracking of Unit*n*.

**Figure 1:**
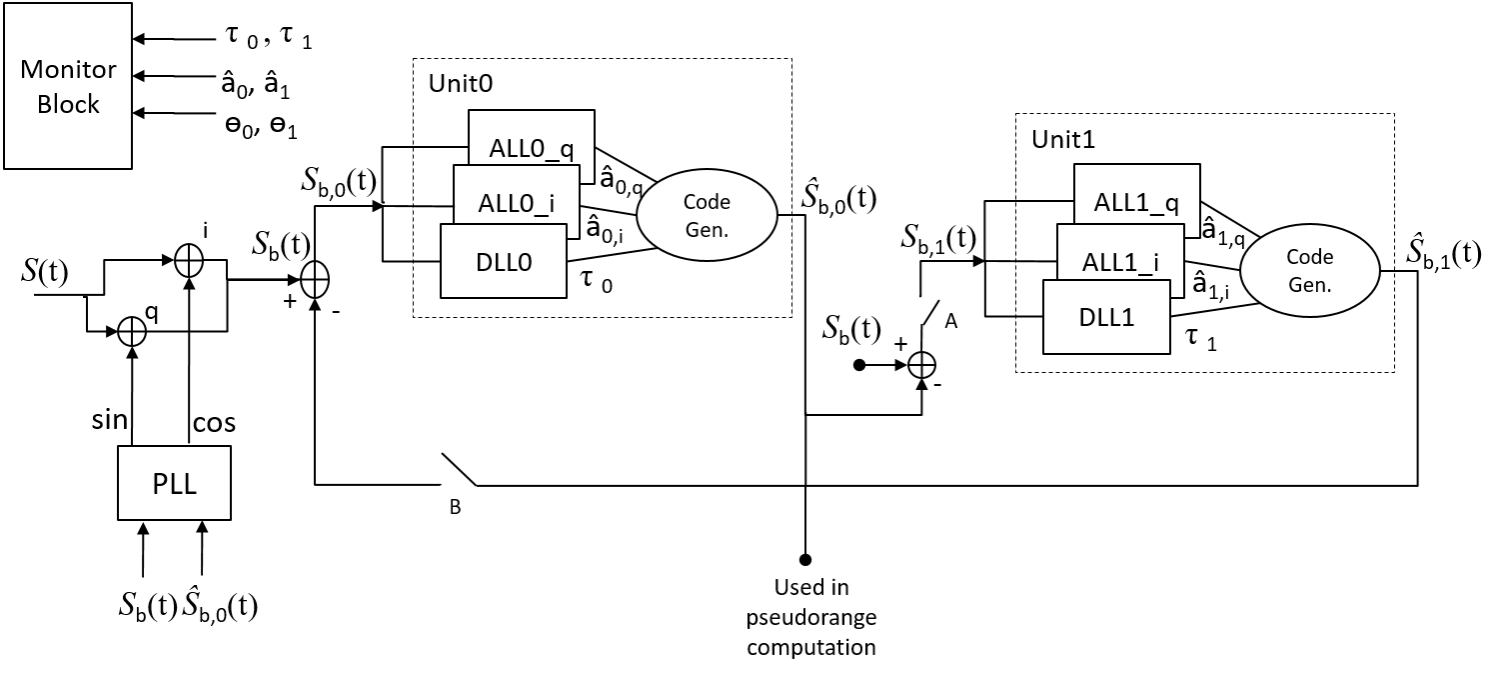
Extended Coupled Amplitude Delay Lock Loop (ECADLL) architecture for a single GNSS channel and configured for spoofing detection.

From (3) and Figure 1, the working principle of the ECADLL can be understood. Using a feedback loop, it subtracts from the overall received signal the sum of the estimated signals in other units. In this way, the structure is able to separate the impairment component from the satellite signal and track each one on a different units.

As can be observed in Figure 1, the total structure for one GNSS channel has a single Phase Lock Loop (PLL), plus one unit for each additional signal that has to be tracked. In the case considered in this work, the structure will have two units, one for the Line Of Sight (LOS) signal coming from the satellite and the other to track the spoofing signal.

Inside each unit, a DLL using a narrow correlator is used to estimate ${\hat{\tau }}_{n}$ and uses the normalized dot-product discriminator:

(4) $$d\tau =\frac{I_{D}\cdot I_{P}+Q_{D}\cdot Q_{P}}{S_{P}}$$

where $I_{P}$ and $Q_{P}$ are the prompt correlations of the in-phase and quadrature signals, respectively. $S_{P}=I_{P}^{2}+Q_{P}^{2}$ and $I_{D}$ and $Q_{D}$ are the early-minus-late of the I and Q channels.

Inside the ALL, the amplitude ${\hat{a}}_{n}$ and phase ${\hat{\theta }}_{n}$ are estimated as:

(5) $$\begin{array}{c} \left.{\hat{a}}_{n}=\sqrt[{}]{{\hat{a}}_{n,i}^{2}+{\hat{a}}_{n,q}^{2}}\right.\;\;\;\;\;\;\;\;\;{\hat{\theta }}_{n}=tan^{-1}\left(\frac{{\hat{a}}_{n,q}}{{\hat{a}}_{n,i}}\right) \end{array}$$

where ${\hat{\theta }}_{n}$ is the estimation of $\theta_{0}+\theta_{n}$. Amplitude values, ${\hat{a}}_{n,i}$ and ${\hat{a}}_{n,q}$, are estimated using the in-phase and quadrature results of the prompt correlation $I_{P}$ and $Q_{P}$, as:

(6) $$\begin{array}{c} {\hat{a}}_{n,i}=\frac{I_{P}}{\lambda } \end{array}$$

(7) $$\begin{array}{c} {\hat{a}}_{n,q}=\frac{Q_{P}}{\lambda } \end{array}$$

where *λ* is a damping factor, slightly smaller than one, used to adjust the estimation accuracy.

The PLL structure in Figure 1 does not use the same correlation output as other units. It uses the correlation values between the total incoming signal (s(t)) and the Unit0 local code (c(t − $\tau_{0})$). Thus, in the presence of impairments, the local carrier phase will not be completely aligned with the carrier phase of the satellite signal. Nonetheless, the carrier phase error will be estimated by the couple of ALLs inside each unit. Actually, recovering the relative phase shift for any single signal component will be necessary, in order to produce the correct replica and add it to the feedback signal.

**Figure 2:**
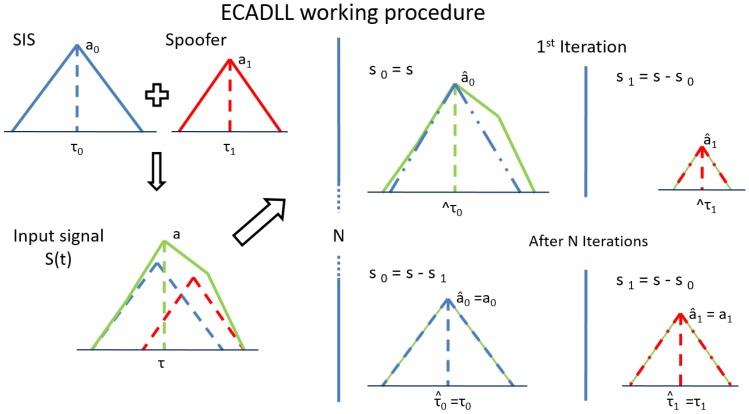
ECADLL basic working procedure when the satellite signal and the spoofing signal are aligned in phase. On the left, the incoming signal is shown, and on the right, the solution after N iterations. Only the in-phase channel is shown in this image for simplicity.

As an example, in Figure 2, the basic working procedure of the ECADLL algorithm is shown when the spoofing and satellite signals are aligned in phase. On the left side of the picture, an estimated correlation function of a generic received signal affected by spoofing is shown. On the right part, we observe the results for the first iteration and for the iterations after the architecture reaches the steady state. At the beginning of the tracking procedure, the architecture starts tracking the residual of the operation s(t) − ${\hat{s}}_{0}$ inside Unit1. At the following iteration, the feedback Switch B is closed, and after a transition time, the two units’ estimations will be locked at values very close to the originals, such that ${\hat{s}}_{0}\approx s_{0}$ and ${\hat{s}}_{1}\approx s_{1}$.

**Figure 3:**
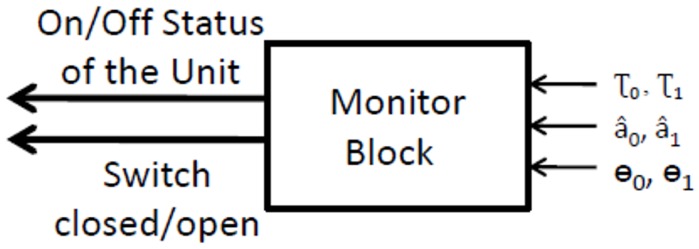
Monitoring block for ECADLL. It receives as inputs the estimations of delay, amplitude and phase, and it controls the powering and insertion of units inside the loop.

A monitoring block, shown in Figure 3, receives as inputs the estimations of ${\hat{\tau }}_{n},{\hat{a}}_{n}$ and ${\hat{\theta }}_{n}$, for all of the units, and it is in charge of turning on and off each of them. We worked under the assumption that a spoofing attack will happen after some time that the receiver is turned on, so the monitoring block is in charge of deciding the moment when each unit is to be introduced by closing Switch A.

If the spoofer is not present, Unit1will be tracking noise, so its amplitude ${\hat{a}}_{1}$ will be small, and its delay ${\hat{\tau }}_{1}$ will wander randomly. Once the spoofer signal appears on the satellite signal correlation space, Unit1 will quickly track the interference signal, thus increasing its value of amplitude and locking the DLL around a value of $\tau_{1}$. At this moment, the monitoring block will close Switch B, starting the feedback loop and obtaining a better estimation of the parameters.

The thresholds used by the monitoring block are defined based on the product of front-end bandwidth (*B*) and the time of chip ($T_{c}$). The product $B\cdot T_{c}$ affects the shape of the correlation function, rounding the peak of the correlation when the product is small. This effect causes a degradation on the detection capabilities, for impairments that are close to the peak.

Section 4 discusses the spoofing detection algorithm, which uses as inputs the estimations of each unit, i.e., ${\hat{\tau }}_{n}$, ${\hat{a}}_{n}$ and ${\hat{\theta }}_{n}$, and takes the decision of whether an impairment is present in the incoming signal or not.

## 4. Using ECADLL Information for Spoofing Detection

**Figure 4:**
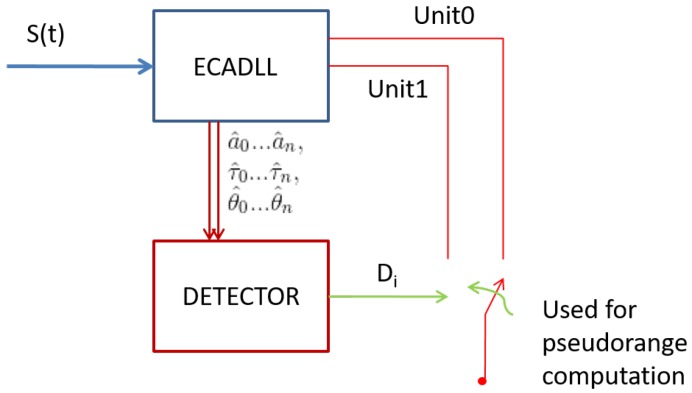
Decision making block diagram.

The ECADLL architecture was originally proposed as a multipath mitigation technique. It is able to eliminate the tracking errors under multipath scenarios for signals having a delay larger than ≈50 ns relative to the satellite signal [12]. The goal of this work is to study and propose a reliable detection technique, able to recognize the spoofer’s presence. In Figure 4, a basic block diagram is presented, where $D_{i}\left(t_{k}\right)$ is the decision variable for the *i*-th channel at time $t_{k}$. Its values are defined as:

(8) $$D_{i}\left(t_{k}\right)=\left\{\begin{array}{cc} 2 & \mathrm{Spoofer}\\ 1 & \mathrm{Impairment}\\ 0 & \mathrm{No}\;\mathrm{impairment} \end{array}\right.$$

It is important to notice that the information provided by the ECADLL architecture is soft information on the estimation of the delay, amplitude and phase of the different signals (${\hat{a}}_{0}\ldots {\hat{a}}_{n},{\hat{\tau }}_{0}\ldots {\hat{\tau }}_{n},{\hat{\theta }}_{0}\ldots {\hat{\theta }}_{n}$). This information is combined to be used as an effective spoofing detection method and has the potential to help with estimating the real user position under spoofing attacks.

### 4.1. Detecting a Generic Impairment

The first thing the algorithm should focus on is detecting a generic impairment. Just by having this information, the receiver is put into an alert stage, and it does not trust the channels that are flagged as impaired.

In order to detect that an undefined impairment signal is present, either multipath or spoofing, an initial decision can be made using the basic information of Unit1. If the DLL in Unit1 is locked, thus having a low variance of ${\hat{\tau }}_{1}$, and the relative amplitude ${\hat{a}}_{1}/{\hat{a}}_{0}$ is greater than a certain threshold ${\hat{a}}_{th}$, the channel can be declared as impaired, and the error mitigation for multipath starts working.

(9) $$D_{i}\left(t_{k}\right)=1\;\mathrm{if}\;\sigma_{{\hat{\tau }}_{1}}<\sigma_{th}\;\mathrm{and}\;\frac{{\hat{a}}_{1}}{{\hat{a}}_{0}}>a_{th}$$

where $\sigma_{{\hat{\tau }}_{1}}$ is the variance of the estimated delay ${\hat{\tau }}_{1}$ in a 1-s window. A window of 1-s duration was used, as a tradeoff providing enough data points to have a good estimation of the variance $\sigma_{{\hat{\tau }}_{1}}$, but keeping the capability to make a decision at a good rate. Thresholds $\sigma_{th}$ and $a_{th}$ are defined according to the DLL bandwidth and the expected level of noise. In order to assess the threshold $\sigma_{th}$, we observed the relationship between the variance of the DLL when tracking a simulated interference signal and the variance when no additional signal was present. Using these values, we were able to obtain an appropriate threshold for the specific configuration. For threshold $a_{th}$, we empirically chose to flag any signal with at least 10% of the Unit0 signal power, since any signal stronger than that is likely to be an interference signal.

**Figure 5:**
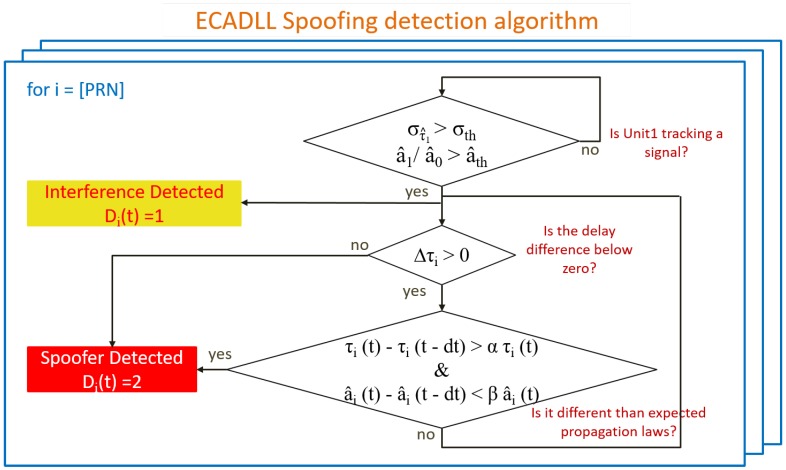
Figure of the proposed ECADLL algorithm. The procedure is repeated for all of the satellites, and it is able to classify the signal tracked by Unit1 as either impairment or spoofing.

In Figure 5, we can observe how the top-most decision is the discrimination between classifying the signal as impairment and no impairment. Summarizing, the channel will be flagged as impairment if the tracking of Unit1 is considered locked to a signal.

### 4.2. Classification as Spoofer Presence

The generic impairment detection is the first step of the detection algorithm. Once it has been classified as impairment, the goal is to recognize whether or not it can be labeled as a spoofing attack. In order to effectively declare the impairment as a spoofing signal, different aspects need to be taken into account.

#### 4.2.1. Detection of Negative Delays

A simple check that can be performed in order to decide whether or not a spoofer is present is to observe if the relative delay is negative, such as:

(10) $$\hat{\delta }\tau =\tau_{1}-\tau_{0}<0$$

In this case, a spoofing attack can be declared safely assuming that the Unit0 is tracking a non-zero level satellite signal. Given the physical nature of the multipath and assuming that the satellite signal has a non-zero power level in the receiver, it is not possible for a multipath reflection to have a delay smaller than the LOS signal, given that, by definition, the LOS is the minimum distance between the receiver and satellite.

On the other hand, a spoofing signal could perfectly arrive into the receiver before the satellite signals, thus giving away its presence. A spoofer could avoid this by managing its delay correctly, but this simple check is a first hard detection to exclude these cases. Following this first discrimination, the impairment will always be declared as a spoofer when (10) is true, and we can observe in Figure 5 how it is implemented as a second level of discrimination.

#### 4.2.2. Detection by Using the Physical Laws of Propagation

One of the main differences between the spoofing signal and the multipath signal is that the latter is bounded by the physical laws of propagation. Due to these physical limitations, it is not possible for the multipath signal to increase its relative delay ${\hat{\tau }}_{1}$, while maintaining the same amplitude ${\hat{a}}_{1}$. This is due to the fact that, in order to have a longer delay, the satellite signal needs to travel a longer physical path, and as a result, its power level will decrease. This consideration means that a single multipath signal will always have a smaller amplitude when the delay is larger. These bounds do not apply to spoofing signals. This remark is also valid the other way around, where a closer delay is bounded to have a greater amplitude.

A spoofing attack may be able to replicate the signal propagation behavior of the signal when it attempts to align the spoofing signal with the satellite one. Nevertheless, when the spoofing signal takes control of the receiver and the push-off phase starts, the satellite signal will be present in the correlation domain, and it will be bound to the physical laws. The satellite signal in the correlation domain will have the same amplitude regardless of the relative delay to the spoofer.

This can be defined for time $t_{k}$ and the i-th channel if:

(11) $$\begin{array}{c} \;\mathrm{if}\;\tau_{1}\left(t_{k}\right)-\tau_{1}\left(t_{k}-dt\right)>\alpha \tau_{1}\left(t_{k}\right) \end{array}$$

(12) $$\begin{array}{c} \;\mathrm{and}\;a_{1}\left(t_{k}\right)-a_{1}\left(t_{k}-dt\right)<\beta a_{1}\left(t_{k}\right) \end{array}$$

where dt is the time difference between the two measures. Coefficients *α* and *β* are smaller than one and are defined using the expected propagation law. When the conditions (11) are met, the decision for spoofing attack is made, i.e., $D_{i}\left(t_{k}\right)=2$. In our case, we assumed a conservative linear decay, so both *α* and *β* are defined as 10%, meaning that a 10% increment in delay will require at least a 10% decay of the amplitude in order to not declare it as a spoofer. Once a channel is declared as being spoofed, it will maintain its status as long as the DLL in Unit1 is locked. It is important to mention that these statements hold as long as the units are tracking continuously the signal between times $t_{k}-dt$ and $t_{k}$. If for any reason the Unit1 loses track of the signal, then the check is not valid anymore.

**Figure 6:**
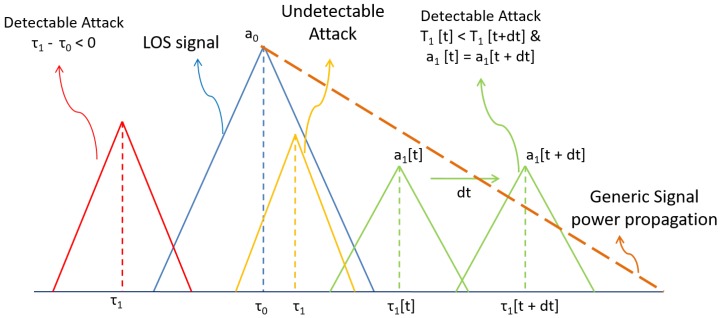
Impairment distinction based on soft observations. The signal in red is detected as a spoofing attack because the delay difference $\tau_{1}$ − $\tau_{0}$ is negative. The green one is detected because the delay increases, but the amplitude is maintained constant, and the yellow one is classified as impairment because there is not enough information to allow discrimination.

In Figure 6, all of the different regions of the decision are graphically shown. Taking into consideration these behaviors, we can distinguish between generic impairments and spoofing attacks. It is important to notice that these remarks are assuming the spoofer will behave in a specific way, so if the spoofer does not follow these criteria, the receiver will be only able to label it as a generic impairment and not a spoofing attack. Nonetheless, the assumptions presented above are common during spoofing attacks, and in the cases where the spoofer is more harmful, it will likely fall into one of the defined regions.

#### 4.2.3. Additional Remarks for Spoofing Classification

One additional remark that can be made is based on the knowledge of the dynamic behavior of the receiver, e.g., checking for the continuity of the impairment signal over time if the user is in a dynamic environment. In dynamic scenarios, multipath rays will be appearing and disappearing as the physical scenario (objects, buildings) changes around the receiver. In these cases, having an impairment signal that is persistent for several seconds is highly improbable, so a spoofing attack could be declared.

On the other hand, in a static scenario where the receiver is fixed, this assumption does not hold anymore, because a reflection from a nearby object can be persistent over time. Nonetheless, in a static case, the receiver can be declared as spoofed once the error in the position, velocity or time surpasses a certain value that a multipath signal will unlikely produce; this assumption only holds if the antenna position is georeferenced. In static cases, it is also important to remember that multipath signals can be observed and classified a priori if the receiver is in a known and fix environment, so anything that is not the known signals can be also classified as spoofing. All of these considerations are heavily case dependent, and that is why they were not included in the general classification algorithm; but they could be effectively used to adapt the algorithm to a given scenario.

### 4.3. Improving Computational Load and Detection Latency

One drawback of the use of basic ECADLL is that when no additional signal is present, Unit1 of each channel is continuously searching for it over random delays. This behavior creates additional computational burden and may cause a delay in the estimation of the real characteristics of the spoofing/multipath signal, because if the delay estimation is wandering randomly, it may take a longer time for it to converge on the right delay once the additional signal is present. In order to improve this situation, in our implementation, we control the turning on/off of the Unit1 detecting distortions on the correlation function using a Ratio Metric (RM).

It has been proven that the SQMT using RM works well detecting distortions in the correlation function [14,33]. Unfortunately, these techniques are not able to estimate the delay of the spoofing signal or mitigate the errors caused by these signals in the position and time solution. Using just one RM to detect distortions in the correlation functions and as an additional input to the monitoring block, the estimation and detection latency of the spoofing signal and the computational load can be improved.

The RM is defined as:

(13) $$M\left(t_{k}\right)=\frac{I_{E}+I_{L}}{mI_{P}}$$

where $I_{E}$, $I_{L}$ and $I_{P}$ are the early, late and prompt correlator outputs of the coherent DLL at a certain time $t_{k}$ and *m* is the slope of the DLL discriminator. The monitoring block computes the mean value of *M* during a 1-s time window, and if it surpasses a pre-defined threshold based on the probability of false alarm, Unit1 is inserted in the feedback loop in order to detect the signal generating the distortion as quickly as possible. A 1-s time window was again chosen as a trade-off to obtain a correct estimation of the mean value and the time needed by the algorithm to make a decision. For spoofing detection, a 1-s time window is still well below the time that a typical spoofing attack will need to gain control of the receiver, which usually extends to several minutes [38].

**Figure 7:**
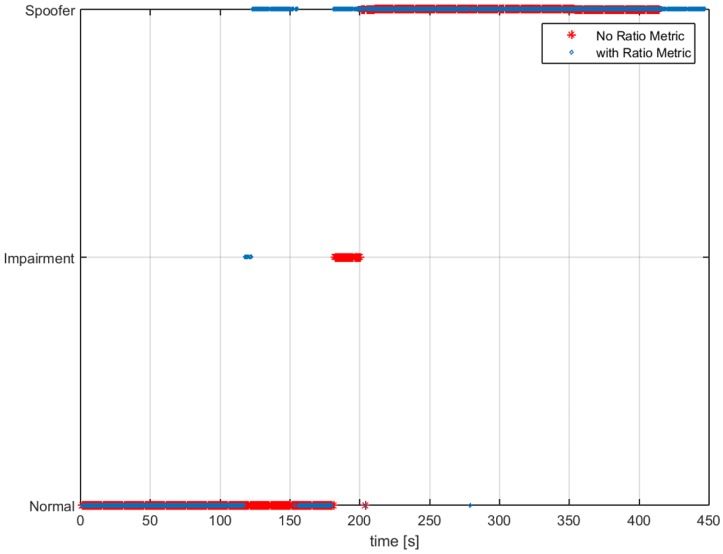
Decision made for a spoofing attack scenario. In red is depicted the decision when no Ratio Metric (RM) is used and in blue when using RMs inside the monitoring block.

**Figure 8:**
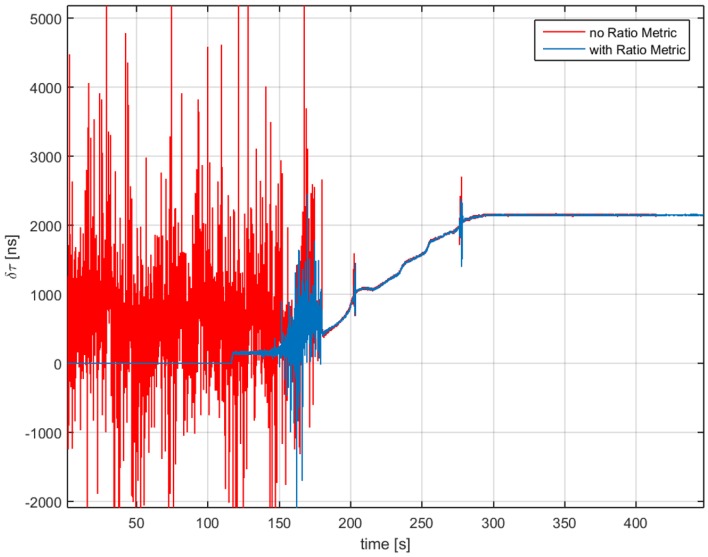
Delay estimation for a spoofing attack scenario. In red is depicted the decision when no RM is used and in blue when using RMs inside the monitoring block.

In Figure 7, we can observe the impact on the detection latency when using the RM. When using RM inside the monitoring block, the spoofing attack is detected as soon as the push-off phase starts, around 120 s. Following this initial detection, the metric has some misdetection, and then, it recovers and declares the spoofing attack for the duration of the test. Furthermore, in Figure 8, we can observe that the delay estimation does not change and is less noisy during the time when the attack starts. Having Unit1 turned off for the first 120 s will greatly help in the computational time for the scenario.

In Section 6.3, we provide numerical and analytic results for the improvement of the computational load and the impairment detection latency.

## 5. Experimental Setup and Initial Results

In order to assess the capabilities of the ECADLL technique under spoofing attack, the Texas Spoofing Test Battery (TEXBAT) was used [38]. The battery consists on a set of high-fidelity datasets that include spoofing attacks in different configurations. The scenarios are based on two clean recordings, free of impairments, one static and one dynamic. Different configurations for the type of attack, relative power to the real signal and targeted variable, either time or position, are distributed between the different scenarios. In Table 1, the basic description of the datasets and the nomenclature used for the remainder of the paper can be found.

Results for TEXBAT ds3 are presented hereafter. This scenario is a static matched-power time push, where the spoofer is inserted after 80 s aligned with the LOS signal, and the initial pull-off is done at around 110 s.

It is important to notice that the results presented here were obtained transmitting the TEXBAT dataset by cable connection to a GPS L1 front-end, named SIGE2 [39], and processing the results using the ECADLL software receiver. By doing so, the signal was filtered through a front-end filter, which has a 4-MHz bandwidth and a resolution of two bits. Furthermore, the sampling frequency is around 16.3676 MHz, and the data were processed at an intermediate frequency of 4.1314 MHz. This configuration provides a lower resolution than the original TEXBAT files, but gives a more common front-end configuration similar to the ones found on commercial receivers.

**Figure 9:**
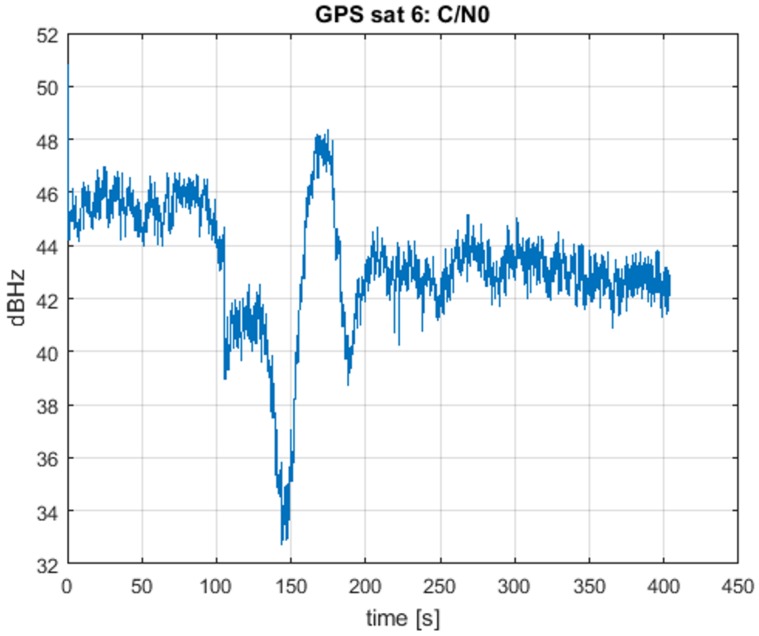
$C/N_{0}$ for Satellite 6 using TEXBAT ds3.

Results are presented for one of the channels, but similar behaviors are obtained for the others. In Figure 9, the estimated $C/N_{0}$ of Satellite 6 is presented. It can be observed that it has a similar trend to the one shown in [38], but with a small loss of power, of a few dB, due to the different front-end configuration and re-transmission.

**Figure 10:**
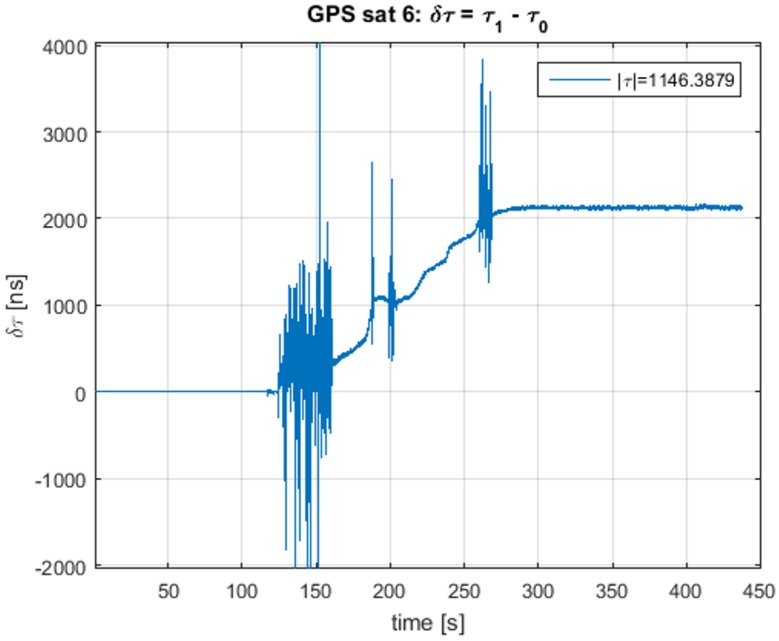
Delay difference between Unit1 and Unit0 for TEXBAT ds3.

In Figure 10, the delay difference $\delta \tau =\tau_{1}-\tau_{0}$ is plotted. In this figure, the working of the ECADLL can be observed: during the first 120 s, the estimated delay is zero, as Unit1 is still turned off. When the asymmetry is revealed, after 120 s, it locks in to the signal that is separated from the spoofing signal being tracked by Unit0. After 100 s of pull-off time, Unit1 estimates the correct delay difference of the real signal as ≈2000 ns, which corresponds to the 600 m of error introduced by the spoofing attack, as stated in [38].

**Figure 11:**
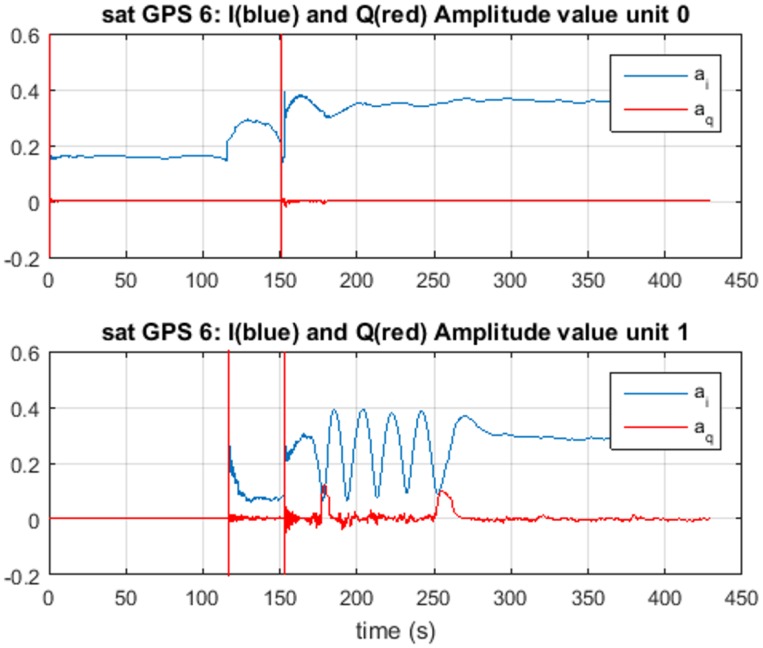
Amplitude estimation for Unit0 (**top**) and Unit1 (**bottom**) for TEXBAT ds3. In blue, the in-phase amplitude estimation, and in red, the quadrature estimation are shown.

**Figure 12:**
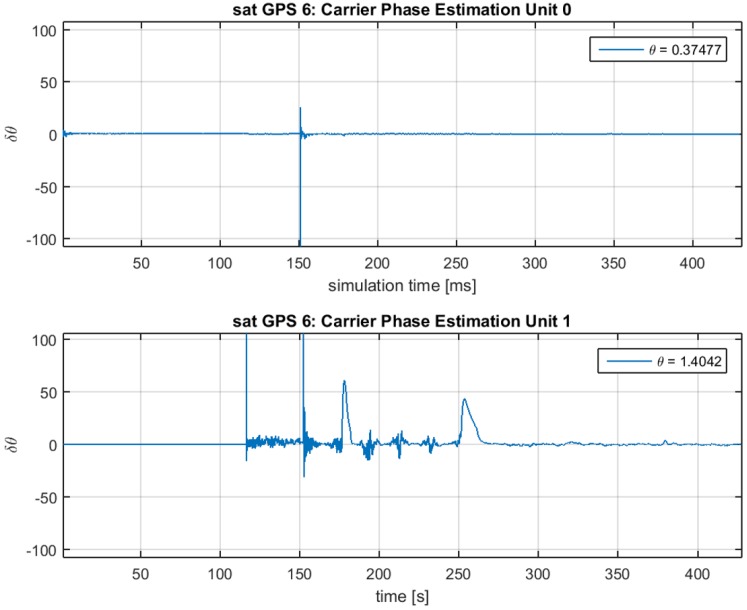
Phase estimation for Unit0 (**top**) and Unit1 (**bottom**) for TEXBAT ds3.

In Figure 11 and Figure 12, the estimated amplitudes ${\hat{a}}_{0}$ and ${\hat{a}}_{1}$ and the estimated phase differences ${\hat{\theta }}_{0}$ and ${\hat{\theta }}_{1}$ are presented for Unit0 on the top panel of each figure and Unit1 on the bottom one. For the amplitude, we observe that the final values correspond to ${\hat{a}}_{0}=0.35$ and ${\hat{a}}_{1}=0.28$; the difference between them is close to the 1.3 dB of spoofing advantage set for the matched-power scenario. Observing these values, we know that Unit0 is tracking the spoofing signal, and Unit1 is tracking the satellite signal.

In this case, given that the detection algorithm as shown in Figure 5 always assumes that the spoofing signal is present in Unit1, the first stage of the detection would fail, but the second step will detect the spoofing signal because the signal processed by Unit1 will not change its power while the relative delay is changing. No matter in what stage of the algorithm the spoofing decision is made, once $D_{i}\left(t\right)=2$, we can assume that the spoofing signal is the one with the higher power, because this is needed to take control of the receiver. In such a case, in order to mitigate the effects of the spoofing attack, information from the correct unit needs to be used for pseudorange computation, as shown in Figure 4, as well as in the PLL of Figure 1, where ${\hat{S}}_{b,0}$ should be adjusted accordingly.

For the spoofing detection, it can be seen that Unit1 starts following the signal that is being pulled-off after it has a δτ of at least 300 ns, at around 150 s of the dataset. Having a small bandwidth of the front-end will cause the detection to have a delay with respect to the time when the spoofer actually starts modifying the position velocity and time (PVT)solution, due to the rounding effect in the correlation function. Nevertheless, as demonstrated in [13], this latency can be improved using a better product between front-end bandwidth and the time of chip ($B\cdot T_{c}$).

These results show the ability of the ECADLL to detect and track the spoofing signal. It is important to notice that given the many possible configurations of spoofing attack scenarios and the many different parameters of the ECADLL architecture, making a statistical analysis in terms of detection probability is a difficult task left for further investigation.

## 6. Results Using the Spoofing Detection Algorithm

After observing the correct working of the ECADLL for the estimation of the signal parameters using the TEXBAT dataset in Section 5, in this section, we present the results for the spoofing detection algorithm, along with a comparison versus another technique. Numerical results highlighting the detection capabilities and the improvement brought by the inclusion of RM are also presented.

### 6.1. Detecting an Evolved Static Matched-Power Time Push Attack

In this section, we present the results obtained processing scenario ds7, which, as explained in [40], is a static matched-power evolved time push attack, based on the clean static dataset, where a spoofer signal is injected after 110 s aligned in phase with the LOS signal. In the following 20 s, the spoofing signal doubles its amplitude and modifies its phase by a 180 degree rotation. In this way, the spoofer slowly takes control of the receiver without the need to synchronize to the navigation data bits [40]. The spoofing signal maintains its status for another 20 s, where it could perform a navigation data bit attack. At 150 s, the spoofer starts the push off phase, increasing the code phase linearly at a rate of 1.2 m per second and decreasing its amplitude until having a similar amplitude as the original LOS signal.

**Figure 13:**
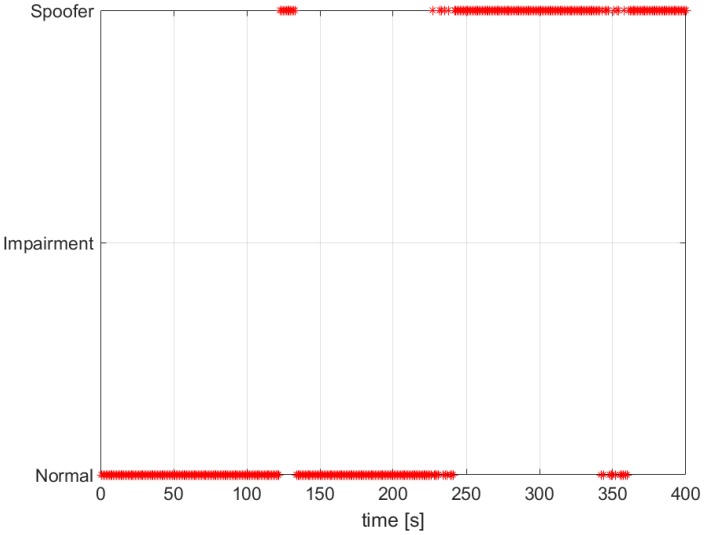
Decision of the spoofing detection algorithm for TEXBAT ds7.

In Figure 13, we observe how the ECADLL plus RM detector is able to detect the spoofing presence around 120 s. After that, it loses the lock and recovers it once the signals are being pulled apart by the spoofing attack.

**Figure 14:**
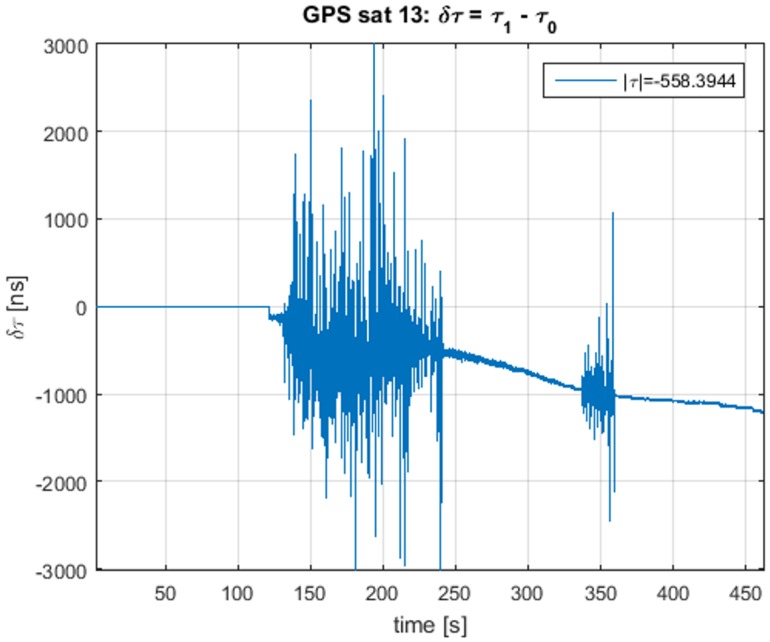
Delay difference estimation for satellite13, using TEXBAT ds7.

**Figure 15:**
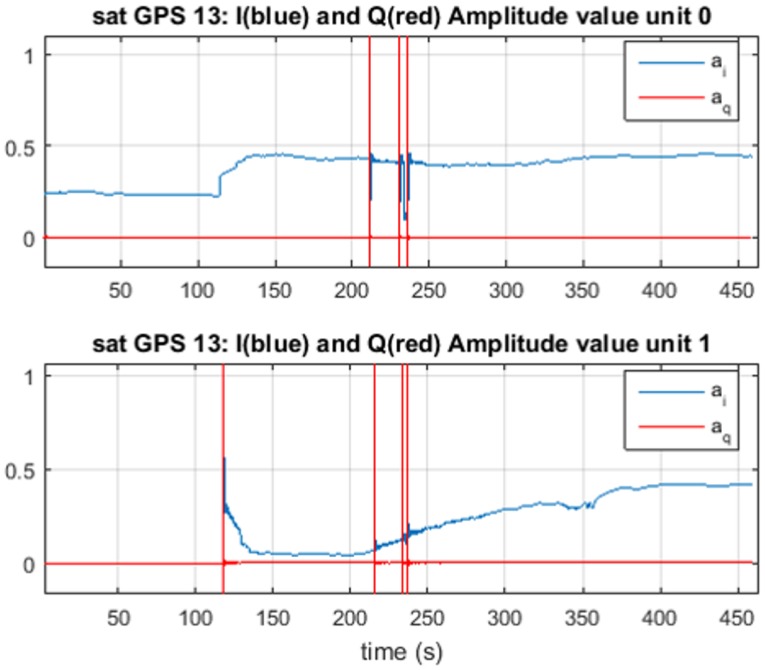
Amplitude estimation for satellite 13, using TEXBAT ds7. In blue, the in-phase amplitude estimation, and in red, the quadrature estimation are shown.

In Figure 14 and Figure 15, we observe the correct estimation of the delay difference $\delta_{\tau }$ and the amplitude estimation that follows the description presented in [40], and we observe how the relative difference in amplitude decreases, as the spoofing signal pushes off the real signal and its modular amplitude is reduced.

### 6.2. ECADLL vs. SQMT

**Figure 16:**
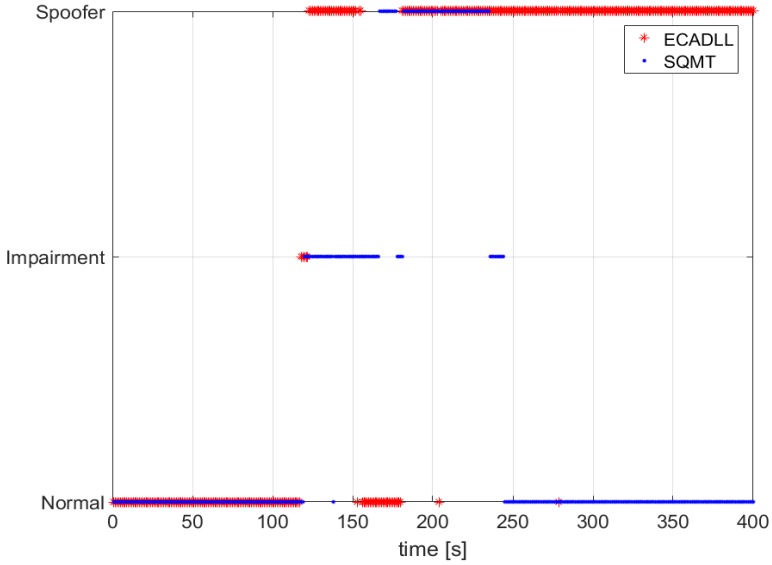
A comparison of the impairment detection using GPS Satellite Number 6 for the static dataset, TEXBAT ds3. In red, the decision made by ECADLL is shown, and in blue, the one using the Signal Quality Monitoring Technique (SQMT).

In this section, we compare the performance of the ECADLL vs. an SQMT as presented in [14]. Figure 16 shows the decision made by SQMT and ECADLL using TEXBAT ds3. We observe that ECADLL is able to detect the distortions at the same time as SQMT given the aid of the RM. ECADLL loses track of the residual signal for some time (160–180 s), and during this time, it is not able to detect the attack. We also observe how the region of detection of the SQMT is limited by the use of fixed correlators, while once the ECADLL recovers the tracking on Unit1, it is able to detect the attack for the duration of the test.

**Figure 17:**
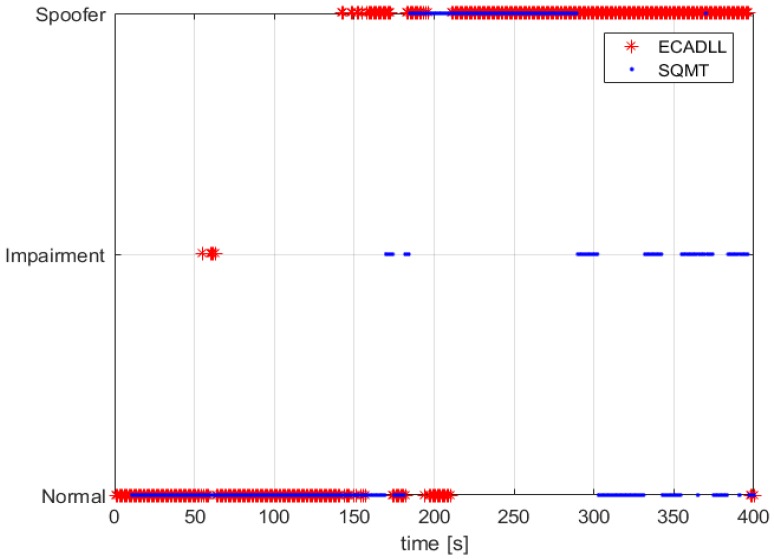
Comparison of the impairment detection using GPS Satellite Number 15 for the dynamic dataset, TEXBAT ds6. In red, the decision made by ECADLL is shown and in blue, the one using SQMT.

In the case of a dynamic scenario, we used TEXBAT ds6, which is similar to ds3, but based on a dynamic dataset recorded by means of a moving vehicle in Austin, TX, and spoofing the position components of the receiver. The detection results, shown in Figure 17, demonstrate that ECADLL has a similar behavior in both static and dynamic scenarios. After 150 s into the test, the architecture reaches the steady state of the feedback and starts tracking the satellite signal on Unit1. The SQMT technique is also able to detect the spoofing attack, but only for a limited duration of the attack, between 170 s and 290 s, similar to what was observed in Figure 16. At around 50 s in Figure 17, we can observe that a small impairment was detected, and we believe this to be a multipath signal present in the dynamic scenarios of TEXBAT.

Comparing the behavior of the two methods in Figure 16 and Figure 17, we observe how ECADLL has similar detection capabilities to the SQMT when the LOS and spoofing signal are close to each other. The ECADLL is able to maintain the detection throughout the test, even when the two signals are well separated, differently from SQMT.

### 6.3. Numerical Results for Detection and Latency

In this section, we provide numerical results for the ECADLL algorithm. These results were obtained by processing four datasets with spoofing signals from the TEXBAT and three different clean datasets, one from the clean scenario in the TEXBAT and the other two obtained by means of a moving vehicle in downtown Turin, Italy. This is by no means a comprehensive set of datasets with all possible configurations of spoofing signals, but we believe that they provide a very insightful initial result for demonstrating the capabilities of ECADLL, the detection algorithm and its future potential.

First, we present a confusion matrix including all of the satellites for the different scenarios that where tested and how they where classified by the algorithm. A confusion matrix is a useful tool to describe the performance of a classification algorithm, such as the one proposed in this paper.

In Table 2, we can observe the confusion matrix and how the different satellites where classified. For the spoofed scenarios, we observed six satellites for each of the four datasets, obtaining a possible of 24 spoofed satellites; the ECADLL algorithm classified 22 of them correctly and missed the detection of two, not detecting correctly one satellite in each of scenarios ds6 and ds7. We believe that these misdetection where caused by the low $C/N_{0}$ that these satellites had for this configuration of the re-transmission, and the algorithm was not able to distinguish clearly the external signal that was present in the correlation domain.

On the other hand, no false alarms where present during the clean scenarios, and none of the satellites were wrongly classified.

Following the confusion matrix, which summarizes the detection capabilities of the technique, we will now focus on the improvements that the introduction of RM brought to ECADLL. Two main features where affected by RM, and they will be defined as Computational Time (CT) and Detection Latency (DL). CT will be defined as the time that the algorithm takes to process a single satellite of one scenario of the TEXBAT datasets, and DL is the time difference between the beginning of the push-off phase of the spoofing signal and the first detection of the algorithm, either as an impairment or spoofer. Of course, these values will change between configurations of the receiver and of the spoofing, computer implementation and characteristics of the datasets. Therefore, they should not be considered as standalone values, but we can obtain useful information if we compare the same dataset while ECADLL is using RM or not. In Table 3, we can observe the numerical improvement for each of the different datasets.

In the Computational Time Improvement (CTI), defined as $CTI=\left(CT_{base}-CT_{RM}\right)/CT_{base}$, we can see how the biggest improvement was obtained when the clean dynamic scenario was used; this means when no spoofer was present. This result is intuitive because when no spoofer is present, the use of RM for the activation of the secondary units will prevent ECADLL from having the two units active at all times. The presence of the Unit1 by itself represents a high burden on the computational load of the receiver, given that it can be comparable to having twice as many channels in tracking and, thus, twice as many correlators.

The scenarios with the presence of spoofing signals do not get that much improvement, because while the spoofer signal is present, both configurations are running two units for each channel. This means that the improvement will be weighted by the amount of time the spoofing signals are not present in the dataset, which for ds3 and ds6 is about 1/4 and for ds7 and ds4 is around 1/3 of the total time.

Eventually, we focus our attention on the Detection Latency Improvement (DLI), defined as $DLI=\left(DL_{RM}-DL_{base}\right)/DL_{base}$. The latency reported here is assuming that the spoofer starts modifying its delay at the time of 110 s of each dataset as reported in [38]. For scenarios ds4 and ds6, which are modifying the position according to their selected pattern, each satellite will be modified with a different delay, unknown to us. According to our analysis, the modification of the delay for ds6 occurs closer to the 110 s than the modification for ds4.

It is interesting to notice the significant improvement in latency obtained when considering ds7. Given the slower rate at which the spoofer modifies the delay with respect to the other scenarios, we get a longer detection time when only relying on the tracking lock of Unit1. When using the ratio metrics, we see that we have a much lower latency, and we are able to flag the presence of impairments in a quicker way.

Values in Table 3 give us the numerical results on how the architecture performance was improved by the introduction of the RM as a simple additional tool for the monitoring block. It is also important to notice that the detection latency is not necessarily referring to a spoofer classification by the algorithm, but it represents the initial warning that the RM gives to the receiver for it to not trust the signal coming from that satellite, until it can be correctly classified or corrected by means of ECADLL.

## 7. Conclusions

In this paper, a spoofing detection algorithm based on the use of ECADLL was introduced. The algorithm is able to identify spoofing attacks when different conditions are met, based on the expected behavior of multipath reflections and on the dynamics of the receiver.

The ECADLL architecture along with the spoofing detection algorithm were tested against the TEXBAT datasets, demonstrating that the technique is able to detect the spoofing attack scenario under certain conditions and that it is able to provide accurate estimation of the parameters of the spoofing signal. The detection algorithm presented in this work works on a single satellite observation. Looking at more satellites simultaneously, using a parallel architecture, can improve the decisions and help identify more easily the spoofing attack.

The ECADLL version presented in the paper, including the spoofing detection algorithm and the ratio metrics in the monitoring block, has overall good detection capabilities and low false alarms, while improving over the original ECADLL in the computational burden and on the detection latency. On the other hand, a careful definition of the thresholds for the ratio metric is important, in order to turn on the units when it is needed and obtain optimal performances.

By only containing two units for each tracked channel, the presented version of the algorithm is only able to provide protection against one spoofing signal at a time. The presence of more than one spoofer competing to gain control of the receiver is quite unrealistic, but other units could be added to the architecture without major modifications, as was presented in [13].

It is important to notice that the anti-spoofing method proposed here is not able to defend the receiver in the cases of overpower attacks, where the vestigial receiver signal is buried under the noise levels introduced by the spoofer. Fortunately, these types of attacks are easily detectable by using in-band power measurements, given that increments of more than a few dBs in the overall power are suspicious and can be a priori excluded.

Solutions for reducing and mitigating the effects of the spoofing attacks in the receiver remain to be explored. This feature could be achieved by switching the navigation solution algorithm to get its ranging information from the unit tracking the satellite signal on a channel under spoofing influence. In order to do this, a powerful and trusted detection algorithm, as the one presented during this work, is a good initial step.

## Figures and Tables

**Table 1:** Texas Spoofing Test Battery (TEXBAT) scenarios description.

Name	Place	Date	Description
clean	Austin, Texas	January 2011	Base scenario for static datasets
ds3	Austin, Texas	January 2011	Static matched-power time push
ds4	Austin, Texas	January 2011	Static matched-power position push
ds6	Austin, Texas	January 2011	Dynamic matched-power evolved position push
ds7	Austin, Texas	June 2015	Static matched-power evolved time push

**Table 2:** Confusion matrix for the two types of datasets processed.

		Predicted Outcome		
		Spoofed	Impairment	Clean
**True**	Spoofed satellite	22	0	2
	Clean Scenario	0	0	18

**Table 3:** Numerical results for improvement of detection delay and computational load. Legend: DL = Detection Latency. CT = Computational Time. DLI = Detection Delay Improvement. CTI = Computational Time Improvement.

	Base ECADLL		ECADLL w\RM		Improvement	
	${\mathrm{DL}}_{\mathrm{base}}$ (s)	${\mathrm{CT}}_{\mathrm{base}}\;($min)	${\mathrm{DL}}_{\mathrm{RM}}\;($s)	${\mathrm{CT}}_{\mathrm{RM}}\;($min)	DLI (%)	CTI (%)
clean	-	35	-	21	-	40
ds3	27	38	17	35	37	7.9
ds6	33	36	17	33	48.5	8.3
ds7	26	41	80	33	57.5	19.5
ds4	93	38	98	30	5.1	21
